# Anaesthetic management in a patient with multiple sclerosis

**DOI:** 10.4103/0019-5049.76598

**Published:** 2011

**Authors:** Lata M Kulkarni, CS Sanikop, HL Shilpa, Anupama Vinayan

**Affiliations:** Department of Anaesthesiology, KLE University’s, Jawaharlal Nehru Medical College & KLES Dr. Prabhakar Kore Hospital, Nehru Nagar, Belgaum, India

**Keywords:** Anaesthetic, demyelination, multiple sclerosis

## Abstract

Multiple sclerosis (MS) is a rare autoimmune demyelinating disorder of the central nervous system clinically manifesting as periodic attacks of varied neurologic symptoms, eventually progressing to fixed neurologic deficits and disability. The treatment is symptomatic and directed towards prevention of future progression of the disease involving multiple agents. We present here a case report of a patient with MS who underwent an orthopaedic procedure under general anaesthesia (G.A.) uneventfully. Anaesthetic implications include assessment of neurological deficits with documentation pre- and postoperatively, awareness towards side-effects, potential drug interactions of medications, selection of suitable techniques/anaesthetic agents, neuromuscular monitoring-guided titration of non-depolarizing blocking agents with lowest necessary dose and avoidance of hyperthermia along with temperature, haemodynamic and respiratory monitoring. Lower concentrations of local anaesthetic (LA) should be used for regional blocks keeping in mind the susceptibility of demyelinated neurons, towards LA neurotoxicity. To the best of our knowledge, this is the first report of anaesthetic management of MS in India.

## INTRODUCTION

Multiple sclerosis (MS) is an autoimmune demyelinating disorder of the central nervous system (CNS) with genetic predisposition characterized by a wide variety of neurological impairment and symptoms due to multi-focal areas of inflammation and demyelination in brain and spinal cord.[1,2] The treatment is symptomatic and with immunosuppressant drugs as there is no cure for MS.

Prevalence, increases with latitude, is rare in Asian countries, highest in north Scotland, northern Europe, and northern United States and in Canada.[1]

Exact prevalence in India is not available, but occasionally an anaesthesiologist may encounter a case of MS.

## CASE REPORT

A boy of sixteen years who was a known case of MS for past eight years presented with fracture shaft of femur following a sudden fall while on treatment of MS following a relapse and was scheduled to undergo open reduction, internal fixation. He had history of limb weakness, relapsing--remitting type with movement-induced muscle spasms, gradually progressing in all the four limbs. During past two months there was diminution of vision in both the eyes and he was restricted to bed. Bowel and bladder function was normal. There was no history of seizures, difficulty in speech, swallowing and breathing. On examination he was moderate in built, weighed 38 kg with normal pulse, blood-pressure, respiration and temperature.

CNS examination revealed a conscious patient with normal higher functions, but mentally depressed. Speech was normal. There was loss of power in all the four limbs, 2/5 in both upper limbs and 2/5 in right lower limb (left lower limb with fracture) as per Medical Research Council (MRC) rating. Co-ordination was impaired in upper and lower limbs with marked movement-induced spasticity, hyper responsive deep reflexes and up-going Babinski. He also had optic neuritis. Gait could not be tested. His sensory system, other cranial nerves, brain-stem function appeared normal. Heart-rate response to deep breathing was normal. His other systems were normal.

Laboratory investigations (routine haematological, liver and kidney function, serum electrolytes), chest X-ray and electrocardiogram (ECG) were normal.

Magnetic resonance imaging (MRI) scan showed patchy bright signals within the cord on T_2_ -weighed images from C_1_ to T_10_ vertebral levels suggesting demyelination. No focal lesion of brain parenchyma was seen.

He was receiving oral methotrexate and folic acid, oral baclofen and methylcobalamin for last six years. Recently, he was treated with intravenous (I.V.) methyl prednisolone during an acute attack followed by tapering dose of oral prednisolone, during last 6-8 weeks.

### Anaesthetic management

The patient was classified as ASA grade III physical status, and parental informed consent was obtained.

Following preoperative counselling and night premedication with oral alprazolam 0.25 mg, next morning patient was shifted to operation theatre (OT). After I.V. access midazolam 1 mg, inj. glycopyrrolate 0.2 mg, inj. ranitidine 50 mg and inj. hydrocortisone 100 mg were administered. Monitoring included oxygen saturation (SpO_2_), ECG, non invasive blood pressure (NIBP) and end tidal carbon dioxide (EtCO_2_), core temperature from the tympanic membrane. OT temperature was maintained at 22° C. Induction was done with 2.5 mg/kg of propofol and 1.5 *µ*g/kg of fentanyl following preoxygenation and intubated with 7.5 cuffed endotracheal tube using 20 mg of atracurium and neuromuscular monitoring (NM) with Train-of-Four (TOF) ratio. He was maintained on N_2_O in O_2_ (66:33), 0.6-0.8% halothane with controlled ventilation and incremental fentanyl 20 *µ*g along with atracurium top-ups of 10 mg using N-M monitoring. I.V. Ringer lactate about 1200 ml was infused. At the conclusion of surgery neuromuscular block was reversed with neostigmine 0.05 mg/kg and glycopyrrolate 0.4 mg satisfactorily. I.V. inj. ondancetron 4 mg was administered.

Post-operatively SpO_2_, NIBP, ECG and temperature monitoring along with O_2_ supplementation by ventimask was done in the recovery room. I.V. Tramadol 2 mg/kg, six hourly was used for post-operative analgesia. There was a rise of temperature 1°C, eight hours postoperatively which was treated with I.V. Paracetamol, 10 mg/kg, and cold sponging promptly. Also there was respiratory distress after twelve hours which subsided after increasing O_2_ concentration to 60%. He was discharged after 10 days. There was no neurological exacerbation of the disease.

## DISCUSSION

MS occurs in genetically susceptible individuals following an environmental, viral exposure that produces activated autoreactive T cells and cytokines, disrupting the blood-brain barrier.[3,4] The inflammatory process produces demyelination and glial scarring (sclerosis) in scattered areas of brain and spinal cord that leads to conduction blockade along the neural pathways wherein Na^+^ channels are affected by endogenous oligopeptides resembling blockade by local anaesthetics LA.[4] This results in multiple sensory deficits, cranial nerve palsies, limb weakness, paraesthesias, cardiac dysrhythmias, autonomic dysfunction, ventilatory disturbances leading to hypoxaemia and respiratory failure.[1–3]

Clinically, there are three main type:

- Relapsing-remitting: episodic symptoms with remissions
- Primary progressive: progressive neurologic deterioration without remissions.
- Secondary progressive: chronically progressive with remissions

All the types can eventually develop into a severe progressive course, ultimately leading to fixed neurologic deficits and disability. Pregnancy is associated with improvement in symptoms and relapse can occur in early post-partum months.[2,3]

Treatment consists of[2,3]

1. During acute attacks, mainly with corticosteroids
2. Immunosuppressants/Immunomodulators to prevent progression. Glatiramer acetate mimics structure of myelin, serves decoy for antibodies. Interferon-β leads to immunomodulation.
3. Symptomatic:
  Paroxysmal pain with carbamezepine, phenytoin, gabapentin.
  Spasticity with baclofen, diazepam, dantrolene.
  Depression with antidepressants.
  Bladder and bowel disturbance with anticho-linergics.

Currently Schwann cell transplantation is under investigation.[2]

Table 1 shows the clinical picture in MS with its anaesthetic implications.[1–3]

**Table 1:** Anaesthetic implications of multiple sclerosis

Demyelination affecting	Clinical signs and symptoms	Anaesthetic implications
Brain	Depression, fatigue, painful seizures, pain syndromes, sensory deficits	Interaction with antidepressants, anticonvulsants agents used for treatment of pain
Corticospinal tracts	Upper motor neuron type of paralysis with spasticity, hyperactive deep reflexes, upgoing Babinski	Upregulation of acetylcholine receptors, altered response to muscle relaxants: N-M monitoring
Brain-stem, optic tracts, cranial nerves	Visual-impairment, nystagmus, diplopia, trigeminalneuralgia, dysarthria, dysphagia, depressed pharyngeal, laryngeal reflexes	Interaction with pain medications used for trigeminal neuralgia, Risk of aspiration— Use of Sellick’s manoeuvre, H_2_ blockers, proton-pump inhibitors, anti-emetics
Brain-stem and spinal cord	Autonomic dysfunction with cardiac dysrhythmia, Impaired control of ventilation, reduced response to raised pCO_2_, diaphragmatic paralysis, ventilatory problems due to reduced respiratory muscle strength, limb-weakness, paresthesias, sensory deficits, Pain-medications/Drugs for spasticity	Cardiac dysfunction Hypotension with Inhalational agents, regional techniques with poor response to fluid loading and pressor agents. Hypoventilation, hypoxaemia, apnoea, resp. failurepost-operative O_2_/mechanical ventilation indicated. Cardiovascular, respiratory monitoring essential. Resistance/sensitivity to N-M blockers, N-M monitoring essential
Others	Even 0.5° rise in body temperature can cause exacerbation	Core and surface temperature monitoring

Literature regarding anaesthetic management contains use of general anaesthesia (GA), spinal and epidural techniques. GA and epidural with low concentrations of LA are considered safe.[2–4] Spinal anaesthesia has been implicated in postoperative exacerbation,[5,6] so also epidurals with higher concentrations and longer duration.[2,3] The demyelinated neurons appear susceptible to the neurotoxicity of LA and aggravate the conduction blockade.[4–6] As regards conduct of G.A no particular agent among induction agents/inhalation agents is preferred.[2,3] Succinylcholine is best avoided as it can produce hyperkalemia due to denervation sensitivity by upregulation of acetylcholine receptors.[2,3]

The latter can also cause resistance to non-depolarizing blocking agents (NDBA).[7] Sensitivity to NDBAs can also be present due to muscle wasting and use of medications such as baclofen, dantrolene[3,8] [Table 1]. N-M monitoring is recommended with titration of the NDBA as required for the surgery.[2,3] Even a 0.5°C rise of temperature can slow the conduction along the demyelinated segment[2–4] resulting in relapse, hence maintenance of O.T temperature and temperature monitoring is essential.

In spite of prolonged periods of immobility, MS patients surprisingly showed the absence of thrombophlebitis and pulmonary embolism (PE) in contrast to other immobile patients suffering from stroke, quadriplegia and flaccid paraplegia.[9] The presence of lower extremity spastic disease in MS patients appears to protect them from thrombophlebitis and PE, as the occurrence of muscle spasms could prevent venous stasis.[9]

Cerebral venous thrombosis (CVT) has been reported in MS exacerbation patients who underwent lumbar puncture (L.P) and were receiving I.V. methylprednisolone.[10] Following L.P, high dose corticosteroids are considered as risk factors for CVT and prophylactic anticoagulant treatment may be warranted when other risk factors of CVT also exist.[10]

As MRI scan of our patient showed involvement of cervical and upper thoracic segments, to detect autonomic dysfunction preoperatively we tested heart rate response to deep breathing. Pulmonary function tests and blood-gases are recommended in the case of significant respiratory embarrassment.[3] As diminution of respiratory muscle strength cannot be ruled out even without symptoms vigilance during postoperative period is essential.[3,5]

Perioperative steroid supplementation to avoid adrenal suppression has been advocated in patients with recent steroid usage[3] and we used I.V. hydrocortisone 100 mg just before induction as our patient had received steroids recently.

Our patient requested for G.A and induction with propofol-fentanyl provided smooth intubation in spite of 65-70% TOF ratio by ED90 dose of atracurium indicating resistance. Intraoperatively TOF ratio-guided top-ups of atracurium provided adequate NM block required for the orthopaedic procedure. Maintenance of O.T temperature to 22°C helped in maintaining normal core temperature. Postoperatively we treated rise of 1°C of body temperature promptly and no relapses occurred in our patient. The postoperative respiratory distress was successfully treated with oxygen therapy by ventimask.

The perioperative period was thus uneventful. To summarize, the optimal anaesthetic management of MS requires careful preoperative assessment, awareness towards perioperative care and postoperative exacerbations of MS. The latter invites special attention and recovery room care with appropriate monitors, oxygen therapy/mechanical ventilation is necessary.

## References

[1] Harser SL, Goodin DS (2008). Multiple sclerosis and demyelinating diseases. Harrison’s Principles of Internal Medicine.

[2] Dierdoff SF, Scott Walton J, Barash PG, Cullen BF, Stoelting RK (2006). Anesthesia for patients with rare and co-existing diseases. Clinical Anesthesia.

[3] Dorotta IR, Schubert A (2002). Multiple sclerosis and anesthetic implications. Curr Opin Anaesthesiol.

[4] Perlas A, Chan VW (2005). Neuraxial anesthesia and multiple sclerosis. Can J Anaesth.

[5] Stoelting RK, Dierdoff SF (2004). Diseases of nervous system in anesthesia and co-existing disease.

[6] Finucane BT, Terblanche OC (2005). Prolonged duration of anesthesia in a patient with multiple sclerosis following a paravertebral block. Can J Anaesth.

[7] Brett RS, Schmidt JH, Gage JS, Schartel SA, Poppers PJ (1987). Measurement of acetylcholine receptor concentration in skeletal muscle from a patient with multiple sclerosis and resistance to atracurium. Anesthesiology.

[8] Bekker A (2005). Neurodegenerative disorders: Surgical procedures and anesthetic implications. ASA Refresher Course Lectures.

[9] Kaufman J, Khatri BO, Riendl P (1988). Are patients with multiple sclerosis protected from thrombophlebitis and pulmonary embolism?. Chest.

[10] Vandenberghe N, Debouverie M, Anxionnat R, Clavelou P, Bouly S, Weber M (2003). Cerebral venous thrombosis in four patients with multiple sclerosis. Eur J Neurol.

